# New ways to use imaging data in cardiovascular research: survey of opinions on federated learning and synthetic data

**DOI:** 10.1093/ehjimp/qyaf012

**Published:** 2025-01-24

**Authors:** Michelle C Williams, Jacqueline A L MacArthur, Ross Forsyth, Steffen E Petersen

**Affiliations:** British Heart Foundation Data Science Centre, Health Data Research UK, Gibbs Building, 215 Euston Road, London NW12BE, UK; British Heart Foundation Centre for Research Excellence Centre for Cardiovascular Science, University of Edinburgh, Chancellors Building, 49 Little France Crescent, Edinburgh EH164TJ, UK; British Heart Foundation Data Science Centre, Health Data Research UK, Gibbs Building, 215 Euston Road, London NW12BE, UK; British Heart Foundation Data Science Centre, Health Data Research UK, Gibbs Building, 215 Euston Road, London NW12BE, UK; British Heart Foundation Data Science Centre, Health Data Research UK, Gibbs Building, 215 Euston Road, London NW12BE, UK; William Harvey Research Institute, NIHR Barts Biomedical Research Centre, Queen Mary University London, Charterhouse Square, London EC1M 6BQ, UK; Barts Heart Centre, St Bartholomew’s Hospital, Barts Health NHS Trust, West Smithfield, London EC1A 7BE, UK

**Keywords:** synthetic data, federated learning, cardiovascular imaging

## Abstract

**Aims:**

Federated learning and the creation of synthetic data are emerging tools, which may enhance the use of imaging data in cardiovascular research. This study sought to understand the perspectives of cardiovascular imaging researchers on the potential benefits and challenges associated with these technologies.

**Methods and results:**

The British Heart Foundation Data Science Centre conducted a series of online surveys and a virtual workshop to gather insights from stakeholders involved in cardiovascular imaging research about federated learning and synthetic data generation. The federated learning survey included 67 respondents: 18% (*n* = 12) were currently using federated learning, 4% (*n* = 3) had previously used it, 31% (*n* = 21) were planning to use it, and 46% (*n* = 31) were neither using nor planning to use it. Highlighted benefits included data privacy and enhanced collaboration, while challenges included data heterogeneity and technical complexity. The synthetic data survey had 22 respondents: 50% (*n* = 11) were currently using synthetic imaging data, 36% (*n* = 8) expressed interest in using it, and 14% (*n* = 3) thought it should not be used. Amongst the respondents, 50% had created synthetic imaging data and 45% had used it in cardiovascular research. Advantages cited included privacy preservation, increased dataset size and diversity, improved data access, and reduced administrative burden. Concerns included potential biases, trust issues, privacy concerns, and the fact that the images were not real and may have limited diversity or quality.

**Conclusion:**

Federated learning and synthetic data offer opportunities for advancing cardiovascular imaging research by addressing data privacy concerns and expanding data availability. However, challenges must be addressed to realize their full potential.

## Graphical Abstract

**Graphical Abstract:**
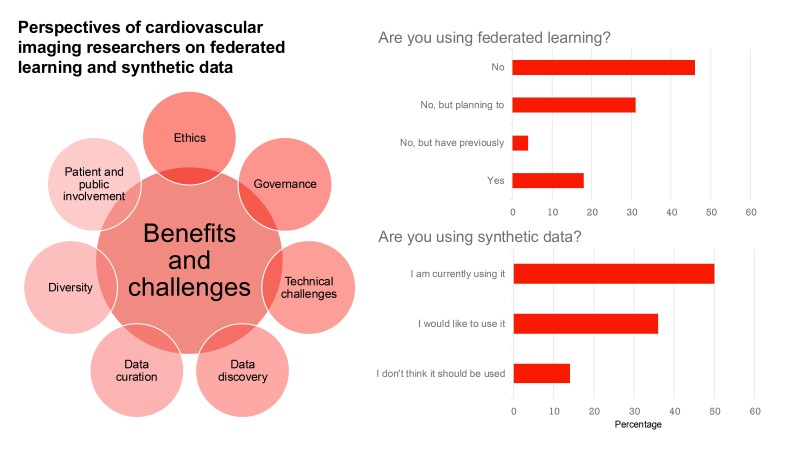

## Introduction

Cardiovascular disease remains the leading cause of death globally,^1^ and in the UK, 7.6 million people are living with heart and circulatory disease.^2^ Cardiovascular imaging research has huge potential to improve patient care. This includes the ability to accurately diagnose cardiovascular disease and guide management, enhance risk stratification, identify early markers of disease, monitor disease progression, and improve our understanding of cardiovascular conditions. However, barriers to the use of imaging data in cardiovascular imaging research include the lack of application of FAIR (findable, accessible, interoperable, and reusable) data principles and lack of linkage to associated healthcare information. This is particularly important for the development of new machine learning tools, which are capable of assessing and utilizing imaging data. Recently, the use of federated learning and the creation of synthetic data have been suggested as alternatives to the creation of large imaging datasets. However, the opinions of cardiovascular imaging researchers regarding the potential of these new techniques have not been explored.

Federated learning is a decentralized machine learning approach that enables model training across separate locations, without the need to share data to a central location. In imaging research, federated learning offers a promising solution for collaborative model training without the need to share large datasets. Each location retains control over its own data, and only model updates are shared and aggregated to refine a global model. Federated learning has shown potential in imaging research, including in the development of machine learning models to assess chest X-rays, magnetic resonance imaging (MRI), computed tomography (CT), and retinal imaging.^3,4^ In research, this could lead to an increase in the size and diversity of datasets, which could improve the generalizability of machine learning models developed for clinical use. However, challenges include potential technical, dataset, privacy, and security issues.

Synthetic data generation involves the creation of datasets, which closely mimic real-world data. This offers a means to expand the diversity and volume of training data available for model development. Synthetic data also holds promise for privacy preservation within imaging research. By generating synthetic images that mimic the properties of real data, researchers can create representations that do not contain identifiable patient information. This synthetic data can be freely shared and used for collaborative research efforts. In research, this could enable a wider range of researchers to access relevant data to develop new tools to improve clinical practice. Synthetic data have shown potential for research involving chest X-rays, MRI, and CT.^5–7^ However, careful validation and calibration are essential to ensure that synthetic data accurately represent the underlying distribution of real images, maintaining the integrity and reliability of research findings.

The British Heart Foundation (BHF) Data Science Centre is a partnership between the BHF and Health Data Research UK, which aims to enable data-led research to improve heart and circulatory health. Through a series of surveys and workshops, we aimed to ascertain the opinions of people involved in cardiovascular imaging research regarding the potential benefits and challenges of federated learning and synthetic data for imaging research.

## Methods

### Study design

We performed a series of online surveys to assess the opinions of individuals involved in cardiovascular imaging research regarding the use of federated learning and synthetic data in cardiovascular imaging research. We also organized an online workshop where the subject of federated learning was discussed in more detail in breakout sessions.

### Participants

Survey and workshop participants were invited if they were involved in cardiovascular imaging research in the UK. They were invited to participate in the BHF Data Science Centre surveys and workshops on improving the use of imaging in cardiovascular research in the UK. They represented a wide range of stakeholder groups, including imaging, cardiovascular, data science, and computer science researchers, National Health Service (NHS) professionals, representatives from NHS organizations, relevant societies, data custodians, and patient/public representatives. For academic participants, invitations were sent to one or more individuals at all academic institutions with active cardiovascular imaging research in the UK, with the option to nominate additional relevant individuals and alternative individuals if they could not attend. Participants included established researchers, early career researchers, post-doctoral researchers, and PhD students.

### Federated learning survey and workshop

We created an online survey (using surveymonkey.com) to gather information from participants in November 2021. We asked them to identify which stakeholder group or groups they belonged to. We asked whether they were using or planning to use federated learning/analysis for cardiovascular imaging research. In addition, participants contributed to a virtual workshop where the advantages and challenges were discussed in break-out groups and opinions were collected using a virtual whiteboard (using mural.com).

### Synthetic data survey

We created an online survey (using surveymonkey.com) to collect the opinions of cardiovascular imaging researchers regarding the use of synthetic imaging data in cardiovascular imaging research in November 2023. Questions included whether they were currently using synthetic imaging data, would like to use synthetic imaging data, or thought that synthetic imaging data should not be used. We also asked whether they had created synthetic imaging data or used synthetic imaging data in research, and if so, what types of synthetic data had they created. We provided free text boxes for them to provide their opinions on the advantages and disadvantages of using synthetic imaging data in cardiovascular imaging research.

### Statistical analysis

Statistical analysis was performed with Microsoft Excel (version 16) and R (R Foundation for Statistical Computing, Vienna, Austria; Version 4.3.0). Categorical data are presented as numbers and percentages. Free text comments from the surveys and workshops were aggregated based on themes.

## Results

### Federated learning

**Figure 1:**
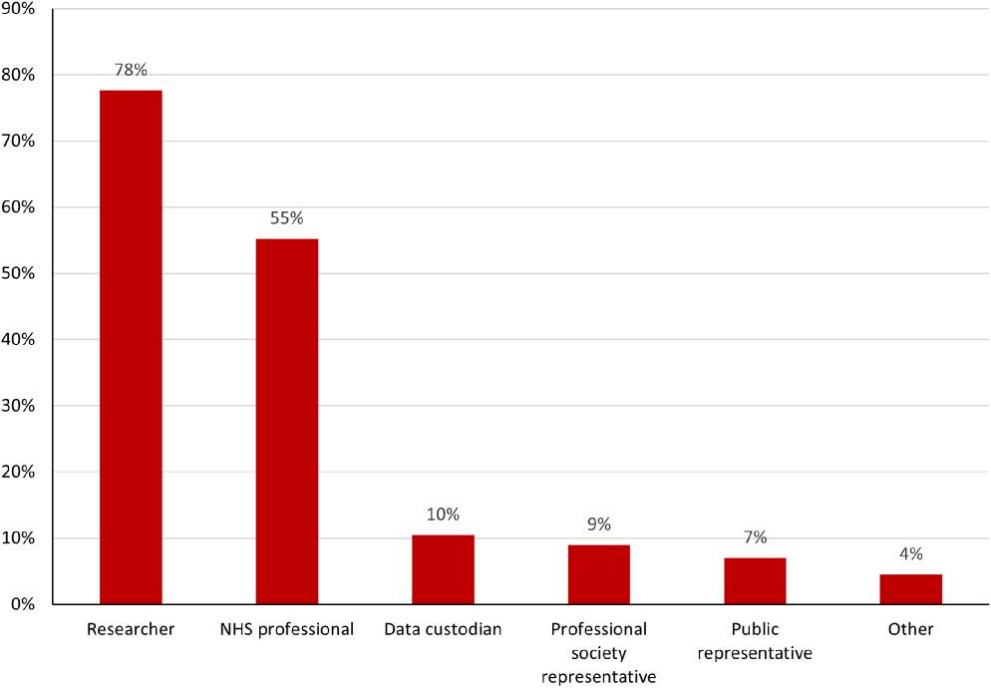
Stakeholder groups of participants in the federated learning survey and workshop.

The survey was completed by 67 respondents (*Table 1*), of whom 78% (*n* = 52) identified as researchers (*Figure 1*). For cardiovascular research, 63% (*n* = 42) used MRI, 38% (*n* = 26) used CT, 33% (*n* = 22) used echocardiography, 27% (*n* = 18) used electrocardiography, 15% (*n* = 10) used positron emission tomography, 13% (*n* = 9) used electrophysiological data, 13% (*n* = 9) used chest X-ray, 9% (*n* = 6) used invasive angiography, and 13% (*n* = 9) used other forms of imaging, including intravascular imaging and retinal imaging. Of the participants, 42% (*n* = 28) were cardiologists, 3% (*n* = 2) were radiologists, and 36% (*n* = 24) were from computing, engineering, mathematics, or data science specialities (*Table 1*).

**Table 1:** Characteristics of survey participants

	Federated learning survey	Synthetic data survey
Number of participants	67	22
Cardiology	28 (42%)	2 (9%)
Radiology	2 (3%)	2 (9%)
Computing, engineering, mathematics, or data science specialities	24 (36%)	8 (36%)
Other	13 (19%)	10 (45%)

Number (%).

**Figure 2:**
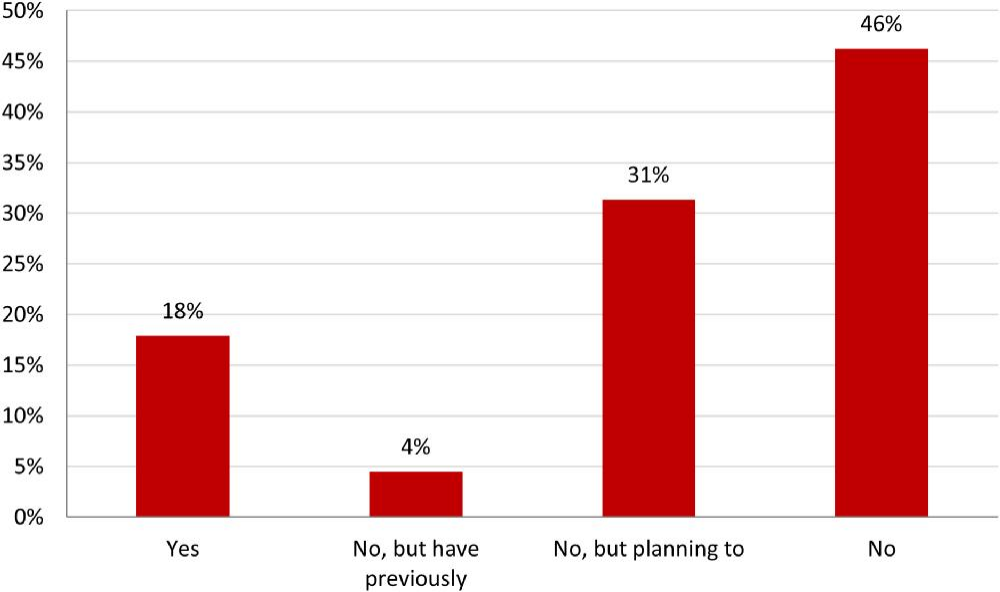
Opinions of survey respondents regarding whether they had used or were using federated learning.

Of the survey participants, 18% (*n* = 12) were currently using federated learning, 4% (*n* = 3) had previously used federated learning, 31% (*n* = 21) were planning on using federated learning, and 46% (*n* = 31) were not using or planning to use federated learning (*Figure 2*). All respondents who were currently using federated learning were based in university academic centres. Amongst the cardiologists, 7% (*n* = 2) were currently using federated learning, 39% (*n* = 11) were planning on using federated learning, 4% (*n* = 1) had previously used federated learning, and 50% (*n* = 14) were not using or planning to use federated learning. Amongst the participants who were from computing, engineering, mathematics, or data science specialities, 17% (*n* = 4) were currently using federated learning, 25% (*n* = 6) were planning on using federated learning, 4% (*n* = 1) had used federated learning in the past, and 38% (*n* = 9) were not planning on using federated learning. Other stakeholder groups were too small to allow sub-analysis.

Participants at the workshop thought that federated learning could be used to enable research across multiple centres or nations, provide access to more diverse data or a wider range of clinical endpoints, increase the available size of the study population, such as for research into rare diseases, and combine cardiovascular imaging data with other data such as routinely collected healthcare information or socioeconomic information.

Opinions on the benefits and challenges of a federated learning approach varied widely amongst participants (*Table 2*). This was particularly apparent for considerations regarding ethics and governance issues, where some participants thought that it would reduce the regulatory requirements and improve access to imaging data. Others thought that it would increase the complexity of approvals to use imaging data in research and create new barriers. Some participants thought that federated learning would reduce technical barriers to research as it would not require a central trusted research environment capable of storing, provisioning, and analysing imaging data. However, others thought that it would increase technical barriers to research as it would necessitate increased local investment in infrastructure and expertise. Participants thought that federated learning would facilitate research on more diverse large-scale datasets, which would particularly benefit research into rare diseases. However, they felt that research involving linked electronic healthcare datasets could become more challenging, and a common format for imaging data with agreed standards would be needed. Diversity in imaging datasets could be increased with federated learning, but participants highlighted that in areas that lacked the technical expertise to implement federated learning tools, this could also create new inequalities in healthcare research.

**Table 2:** Benefits and challenges of federated learning for cardiovascular imaging research

Theme	Benefits	Challenges
Ethics and governance	Data remain with data controller. Potentially reduces regulatory and governance barriers. No data sharing agreements. Models can be shared. Encourages collaborations, including international collaborations. Requires a shift in thinking about how research is performed.	Lack of understanding of techniques and risks involved means that the complexity of approvals might not be reduced. Current regulatory barriers impact scalability. Buy-in from industry is likely to be challenging. Ownership of mutually generated intellectual property is uncertain.
Technical challenges	Does not need a central trusted research environment. Potential for greater transparency in machine learning development. Can be used in multiple centres. No data sharing resources.	Resource and cost implications include time, expertise, infrastructure, and network. Interoperability and risks associated with vendor lock-in. Potential new data security challenges and new safeguards required. New training requirements.
Data discovery and curation	Potential for more agile, reactive, and scalable identification of research cohorts. Potential improvements in research of rare diseases. Access to large scale diverse data.	Working with linked datasets is more difficult. Curation, pre-processing, and quality control are more challenging. Potential duplication of effort at individual sites. Needs data in a common format with agreed standards.
Diversity	Could increase size and diversity of research populations. Could help democratize research by engaging smaller centres. Could be used to test how biases in data affect models. Potential to develop more accurate, fairer, representative machine learning models to support patient care.	Will be challenging to onboard non-research orientated hospitals due to technical and resources requirements. Issue of equity, with academic centres being ready to use these techniques and other centres lacking resources and expertise. May miss patient groups living in areas, which lack resources to implement federated learning.
Patients, carers, and members of the public	Benefits of privacy preservation. Wider range of more representative data	Obtaining and maintaining patient/public engagement and trust. Complicated terms and technologies that need properly explained.

Patient and public contributors highlighted the benefits of privacy preservation and of research involving a wider range of more representative data. However, they also highlighted the importance of obtaining and maintaining engagement and trust of patients and the public. In particular, they felt there was a need for more information for patients and the public, which explained the complicated terms and technologies, which are involved in these types of research.

### Synthetic data

**Figure 3:**
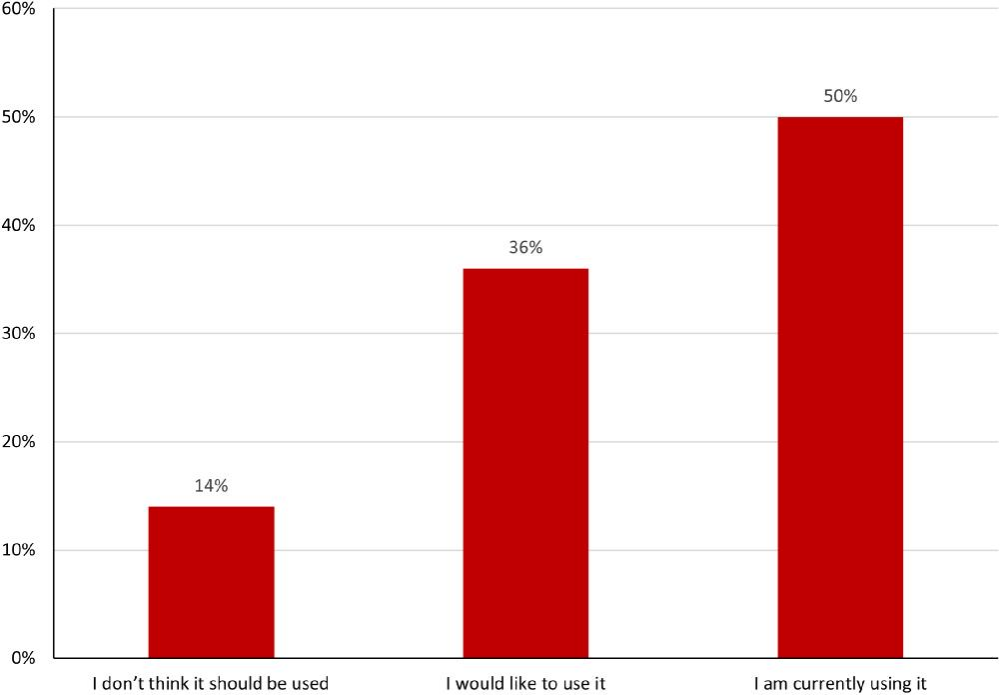
Opinions of survey respondents regarding synthetic imaging data for cardiovascular imaging research.

The survey was completed by 22 respondents, including 9% cardiology (*n* = 2), 9% radiology (*n* = 2), and 36% (*n* = 8) from computing, engineering, mathematics, or data science specialities (*Table 1*). Of the survey participants, 50% (*n* = 11) were currently using synthetic imaging data, 36% (*n* = 8) would like to use synthetic imaging data and 14% (*n* = 3) thought that synthetic imaging data should not be used for cardiovascular imaging research (*Figure 3*).

Synthetic imaging data had been previously created by 11 respondents (50%). The imaging modalities that had been created included CT, MRI, electrocardiogram, electroanatomic mapping, echocardiogram, retinal imaging, and ultrasound. Synthetic imaging data had previously been used in cardiovascular imaging research by 45% (*n* = 10) of the participants. The respondents had used synthetic imaging data for data augmentation for machine learning research and image analysis research and to improve image quality.

Advantages of using synthetic imaging for cardiovascular research (*Table 3*) that respondents highlighted included privacy preservation (14%, *n* = 3), increased dataset size (23%, *n* = 5), increased dataset diversity (32%, *n* = 7), and improved access to data (18%, *n* = 4). Additional advantages that were suggested included potential time savings during the data access process due to reduced governance paperwork, increased availability of data to researchers including early career researchers, and reduced burden on patient volunteers in imaging research (*Table 2*).

**Table 3:** Benefits and challenges of synthetic data for cardiovascular imaging research

Theme	Benefits	Challenges
Ethics and governance	Potential to preserve privacy. Potential time savings for data access.	Potential for private data to be leaked into dataset. New governance frameworks required.
Technical challenges	Commonly used to train deep learning networks. Increased analytic potential. Faster evaluation of machine learning models. No need to perform anonymization. Creating ‘digital twins’ for simulation research. Pre-training machine learning models.	Challenges of creating high resolution datasets. Challenges creating datasets, which are indistinguishable from real imaging data. Potential to introduce new mistakes into the dataset.
Data discovery and curation	Increased size of available datasets. More widely available data. Potential uses for teaching. Improved data access and data sharing. Potential to use for benchmarking of models.	Datasets may not be ‘real’ enough to allow for image analysis or machine learning tasks. Potential issues of trust in the data.
Diversity	Increased diversity of available datasets.	Potential to augment biases in datasets. Potential to amplify errors in datasets. May not be sufficiently diverse to be representative.
Patients, carers, and members of the public	Benefits of privacy preservation. Potential to reduce burden on patient volunteers in research studies.	Concerns about how representative the data is.

Disadvantages of using synthetic imaging for cardiovascular research (*Table 2*) that respondents highlighted included the potential to introduce or augment biases or mistakes (32%, *n* = 7), issues of trust in the resulting models/results (9%, *n* = 2), and the fact that the synthetic images were not real images (32%, *n* = 7). Additional disadvantages included lack of sufficient diversity, the potential to increase errors based on limited data or incorrect modelling, the quality of the resulting synthetic images, privacy concerns with the potential for private data to be contained within the synthetic data, and a current lack of demonstrated use cases outside of machine learning research (*Table 2*). Several respondents questioned whether synthetic data could replace real data and whether it could be representative enough to facilitate the creation of generalizable machine learning models.

## Discussion

In these surveys and the workshop, we explored opinions on using federated learning and synthetic data in cardiovascular imaging research. Federated learning could facilitate research across multiple centres, provide access to more diverse data, increase the size of study populations, and combine imaging data with other datasets. The highlighted challenges of federated learning included the complexity of governance approvals, technical challenges, data curation, and challenges obtaining and maintaining trust of patients and the public. Synthetic data have the potential to increase the size and diversity of datasets, improve access to imaging data for research, and maintain the privacy and security of datasets. The highlighted challenges of synthetic data in research included the potential to introduce or exacerbate biases or mistakes in datasets, the accuracy of the resulting images, and whether the resulting models would be generalizable to real datasets.

Cardiovascular imaging research will benefit from using large numbers of imaging tests from diverse populations. However, there are multiple challenges to the creation of centralized imaging datasets, including the size of the data, privacy concerns, and the ability to share and transfer data between sites or countries. Foundational principles for scientific data management are FAIR.^8^ However, creating centralized imaging resources meeting these requirements is challenging and time-consuming.^9^ The lack of centralized imaging datasets has the potential to hold back cardiovascular imaging research and machine learning research in particular. Federated learning and synthetic data offer potential options to improve the use of imaging data in cardiovascular research. Federated learning could provide increased size and diversity of datasets, which will improve the generalizability of machine learning models to different clinical settings. Synthetic data generation and sharing will increase the range of researchers that can be involved in healthcare research. Together, these tools have the potential to improve clinical practice by enhancing the range and applicability of the resulting research. However, at present, the use of federated learning and synthetic data generation is limited by expertise, cost, and infrastructure requirements. Additional training and investment will be required for these tools to be widely available to non-expert researchers.

Federated learning was proposed by Google as a solution for the local training of machine learning models with aggregation of the training results, but not the underlying data, on central servers.^10^ This is a potentially privacy-preserving technique as the central computers only receive a representation of the initial data, which has been learned by the local models. Federated learning can be used to increase the number of participants in a dataset (horizontal federation) or the types of information available in a dataset (vertical federation). In imaging, research federated learning has been used for research involving various imaging techniques, including brain MRI, prostate MRI, chest X-ray, chest CT, abdominal CT, mammography, thyroid ultrasound, and retinal imaging.^11–18^ Simulation studies have shown that federated learning in imaging research can provide similar results to models trained using shared data.^19^ In cardiovascular imaging research, federated learning provided similar results to centralized learning in a simulated experiment to diagnose hypertrophic obstructive cardiomyopathy on cardiac MRI.^20^ For models to detect hypertrophic cardiomyopathy on electrocardiograms and echocardiograms, federated learning showed improved generalizability compared with models trained using shared data.^21^ However, challenges of federated learning have emerged in these previous studies, including technical, regulatory, and ownership challenges. Different models have the potential to preserve privacy to varying degrees, and issues of biases and mistakes in the underlying data may be challenging to correct unless the resulting datasets are very large. The research groups that were currently using federated learning in our survey were all based in university academic centres. There is potential to perpetuate biases if diverse data are not used for modal training, and expansion beyond large academic centres will be important. There is also the potential to increase health inequalities if advances in technology are only available in certain centres. This includes differences in access between academic vs. non-academic centres, urban vs. rural centres, and socioeconomic variations. Understanding how these factors impact the perceptions of federated learning will also be important. In our study, the opinions of cardiovascular imaging researchers varied widely, with federated learning being seen to be both a benefit and a hindrance to imaging research. In addition, the patient and public contributors highlighted the need for clearer information on what these new techniques involved and their potential risks.

Synthetic imaging data have recently emerged as an option for medical research as an alternative to the direct use of patient data. There are a variety of methods that can be used to create synthetic data, including statistical and machine learning techniques. Federated learning has also recently been used to improve synthetic data generation.^22^ Synthetic data creation exists on a spectrum, from fully synthetic data to synthetically augmented data. Synthetic data are widely used during the internal training of machine learning models, where various image manipulation techniques can be used to increase the variability in the dataset (e.g. rotation, skew, and translation). In imaging research, such synthetic data have been shown to accelerate machine learning model development for chest X-rays.^6^ For cardiac imaging, synthetic data have shown value for pre-training machine learning models for cardiac segmentation on MRI,^23,24^ improving CT image quality,^25^ assessing CT image quality,^26^ and cardiac segmentation on echocardiography.^27^ Utilizing synthetic data is particularly useful when the available training datasets are small or imbalanced.^23,24^ Synthetic data creation may also be quicker to create and share than developing a federated learning infrastructure,^28^ which may have additional benefits such as reduced costs. However, concerns regarding the use of synthetic data include the validity and accuracy of the resulting research and the potential risk of leaking private data into the synthetic data. An important question is whether the findings in a synthetic dataset will be generalizable to real populations.

This manuscript has some limitations, which should be acknowledged. First, the qualitative survey methodology is limited by the range and number of participants. Participants were selected based on their involvement in cardiovascular imaging research within the UK, and the inclusion of other survey participants may have provided different opinions. Second, survey participants represented those with an interest in cardiovascular imaging research and may not be generalizable to other research areas. Third, federated learning and synthetic data are both rapidly developing techniques, and opinions regarding these techniques may also change over time. Fourth, the surveys and workshops were performed between 2021 and 2023, and as this is a rapidly evolving field, opinions of individuals will continue to evolve.

In conclusion, we have provided insight into the opinions of individuals involved in cardiovascular imaging research on using federated learning and synthetic data in cardiovascular imaging research. Opinions varied widely, with both techniques having potential benefits and challenges.

## Data Availability

The data underlying this article cannot be shared publicly as they were collected from participants in a survey, which does not allow for data sharing.

## Lead author biography

Professor Michelle Williams is a professor of Cardiovascular Imaging and Honorary Consultant Radiologist at the University of Edinburgh and NHS Lothian. She is an associate director of the British Heart Foundation Data Science Centre and the Imaging lead. Her research centres around multi-modality non-invasive imaging of the heart and blood vessels, including using machine learning and other advanced analytic techniques. She is the president of the British Society of Cardiovascular Imaging, a member of the executive committee of the European Society of Cardiovascular Radiology, and a member of the Board of Directors of the Society of Cardiovascular Computed Tomography.
